# An Integrated Qualitative and Quantitative Biochemical Model Learning Framework Using Evolutionary Strategy and Simulated Annealing

**DOI:** 10.1007/s12559-015-9328-x

**Published:** 2015-05-03

**Authors:** Zujian Wu, Wei Pang, George M. Coghill

**Affiliations:** College of Information Science and Technology, Jinan University, Guangzhou, 510632 Guangdong People’s Republic of China; School of Natural and Computing Sciences, University of Aberdeen, Aberdeen, AB24 3UE Scotland, UK

**Keywords:** Evolutionary algorithms, Heuristic algorithms, Qualitative model learning, Quantitative model learning, Systems biology

## Abstract

Both qualitative and quantitative model learning frameworks for biochemical systems have been studied in computational systems biology. In this research, after introducing two forms of pre-defined component patterns to represent biochemical models, we propose an integrative qualitative and quantitative modelling framework for inferring biochemical systems. In the proposed framework, interactions between reactants in the candidate models for a target biochemical system are evolved and eventually identified by the application of a qualitative model learning approach with an evolution strategy. Kinetic rates of the models generated from qualitative model learning are then further optimised by employing a quantitative approach with simulated annealing. Experimental results indicate that our proposed integrative framework is feasible to learn the relationships between biochemical reactants qualitatively and to make the model replicate the behaviours of the target system by optimising the kinetic rates quantitatively. Moreover, potential reactants of a target biochemical system can be discovered by hypothesising complex reactants in the synthetic models. Based on the biochemical models learned from the proposed framework, biologists can further perform experimental study in wet laboratory. In this way, natural biochemical systems can be better understood.

## Introduction

Understanding inherent mechanisms and principles in the biochemical systems is one of the main tasks when modelling such systems. To effectively investigate a biochemical system of interest, in silico analysis can be performed to reveal and formalise the underlying cellular functions and biochemical processes. Two different but complementary methods, quantitative and qualitative model learning approaches [3], can be applied to model biochemical systems: a given cellular system can be described and analysed mathematically in a quantitative manner until desired biochemical behaviour is replicated in a virtual cellular environment, for instance, a web-based environment for kinetic modelling and dynamic simulation of cellular networks—WebCell [36]; meanwhile, a biochemical system can be qualitatively modelled and identified through qualitative model learning(QML) [49, 52, 53] when only incomplete knowledge and imperfect data are available. The above facts motivate us to develop an integrative qualitative and quantitative model learning framework, and we expect that by making use of the advantages of both learning approaches better learning performance will be achieved to assist wet-laboratory research.

In quantitative modelling approaches, a dynamic biochemical system is mathematically represented to model molecular mechanisms at a quantitative level, and interactions of molecules may be discovered through such modelling process. In further biochemical analysis and wet-laboratory experiments, more biochemical assumptions may be suggested and verified with the help of such precise quantitative analysis. In addition, cell–cell interactions can also be studied through quantitative simulation or system identification [37].

With the recent development of experimental techniques, large omics data have been made available, and this makes it possible to employ quantitative modelling approaches to analyse the dynamics of biochemical systems at the genomic level and address various biochemical issues. For instance, metabolome, fluxome, transcriptome and/or proteome can all be improved and completed by the use of quantitative approaches. Moreover, topologies of biochemical systems can be identified [29, 65] and parameter values can be numerically determined or estimated [32, 43].

In qualitative modelling approaches, qualitative information extracted from imprecise and incomplete data is used to model real-world problems, which becomes the task of qualitative reasoning (QR) [20, 35]. Continuous aspects of a given dynamic system, for instance, space, time and quantity, can be represented or inferred automatically in QR. In QR-based research, qualitative values, such as, *high*, *medium*, *low*, *zero*, *positive* and *negative*, can be used to describe complicated dynamic systems, instead of using precise numerical values. Therefore, behaviours of target biochemical systems can be predicted and reasoned qualitatively in silico with the support of QR [30].

One of the subfields of QR, qualitative differential equation model learning (QML) [48], involves the construction of qualitative differential equation models of dynamic systems from observed data and existing knowledge. QML has been well developed in the last two decades, and examples of QML systems include MISQ [60], GENMODEL [26], QOPH [15], QSI [10], ILP-QSI [17], and the most recent QML-Morven [47, 50] as well as its scalable version, QML-AiNet [51]. QML is a complementary approach to quantitative system identification [37] and it works well in reasoning dynamic behaviours of biochemical systems, especially when only noisy and sparse experimental data are available. QML can infer plausible qualitative models for a given target biochemical system (for instance, in [70] an integrative modelling approach is studied for stepwise qualitative exploration of biochemical interactions), and these plausible qualitative models could be directly examined by biologists or further refined by quantitative approaches depending on specific research tasks.

In a biochemical system, behaviours of reactants, including the interactions between these reactants, are determined by kinetic laws and concentrations of species. In the presence of abundant quantitative data and sufficient knowledge, it is straightforward to employ sophisticated quantitative modelling approaches and tractable computational tools to first build quantitative models by presuming the model structures and then fit the numeric parameters of these models. However, when available data and knowledge are not enough to assume model structures and perform quantitative analysis, it is essential to use qualitative model learning approaches to first qualitatively infer the model structure and then analyse biochemical systems at a qualitative level [47, 63].

In this research, we propose an integrative framework to explore the biochemical model space at both qualitative and quantitative levels. More specifically, an evolution strategy (ES) [5, 62] is employed in the qualitative approach to perform effective selection and composition of functional modules and heuristically evolve model structures towards the target biochemical systems. Then simulated annealing (SA) [31] is used to quantitatively optimise model kinetic rate constants obtained from the qualitative approach. The motivation of employing ES and SA in our framework is that these two metaheuristics algorithms are suitable for searching qualitative and quantitative model space, respectively, and they have been proven to be effective in similar problems in our previous work [66, 67]. For a comprehensive review of employing ES and SA in biochemical systems identification, the reader is referred to [68], in which the optimisation of model structure and kinetic rates was studied by the hybrid use of ES and SA, respectively. In addition, for a general review of evolutionary algorithms and related issues on their applications, the reader is referred to [46]. It is also worth mentioning that in [22], a co-evolutionary algorithm is used to infer differential equation models of the target system from time series data in a reverse engineering manner.

The rest of this paper is organised as follows. In Sect. 2, biochemical components and models are briefly described and how these components work together are also illustrated. We introduce the background knowledge about the quantitative and qualitative modelling approaches in Sects. 3.2 and 3.1, respectively. In Sect. 4, we present the integrative quantitative and qualitative modelling framework. Some case studies and simulation results with analysis are reported in Sect. 5. In Sect. 6, we compare our system with relevant ones. Finally, Sect. 7 concludes our research with discussions on future work.

## Representation of Biochemical Models

In this research, we reuse the definitions of biochemical building blocks (components) and composition of these components (models), as reported in our previous work [66, 69], to represent the target biochemical system in the form of Petri nets [44]. Components are basic building blocks, and a synthetic model is composed from these components by following a set of composition rules [66]. Definitions of biochemical components and models as well as the applications of composition rules are detailed in our previous work [66, 68]. In this section, a brief introduction about the components and model composition is given as follows.

In general, biochemical components can be defined by Petri nets, which can also be applied to define the two patterns (binding and unbinding) for instantiating components. As shown in Eqs. (1) and (2), the binding and unbinding patterns are defined for further model learning tasks.

1 $$\begin{aligned} P_1+P_2 \mathop {\longrightarrow }\limits ^{k1} P_3 \end{aligned}$$

In Eq. (1), $P_1$ represents a reactant acting as a substrate; $P_2$ denotes a reactant acting as an enzyme; $P_3$ ($P_3=P_1|P_2$) is a complex synthesised from $P_1$ and $P_2$ at a reaction kinetic rate constant $k1$, which is for a synthetic process. It should be noted that in this research we use the symbol ‘$|$’ joining the labels of the two reactants to represent a complex.

2 $$\begin{aligned} P_3 \mathop {\longrightarrow }\limits ^{k2} P_1^*+P_2^* \end{aligned}$$

In Eq. (2), $k2$ is a reaction kinetic rate constant for a disassociation process; Complex $P_3$ is either disassociated to two reactants $P_1^*$ and $P_2^*$, which form the complex $P_3$ itself (an inverse process of the binding pattern presented in Eq. (1), thus $P_1^* = P_1$ and $P_2^* = P_2$), or converted into a new product $(P_1^* \ne P_1)$ and an enzyme $(P_2^* = P_2)$ which is one of the reactants forming the complex $P_3$ described in Eq. (1).

We can take the binding pattern for the instantiation of a component, and in the binding process, two reactants are combined to form a complex reactant. We can also take the unbinding pattern for instantiating a component, and in the unbinding process, a complex reactant is divided into two reactants. In this way, complicated components consisting of more than three reactants can be represented by the composition of components instantiated from these two patterns. As mentioned before, in this research qualitative model, learning with ES is used to evolve model structures generated from these components, and kinetic rate constants associated with the interactions between the reactants in these components are optimised by quantitative model learning with SA.

We here briefly introduce the formation of components: given two sets of reactant labels provided by the user: a set of reactants as species $S_{species}$ and another set of reactants as enzymes $S_{enzymes}$. Each element in $S_{species}$ is selected in turn to be combined with each element in $S_{enzymes}$ to produce a complex and a new reactant, based on the mass-action 1 (MA1) kinetic law [6]. For instance, species $A$ from $S_{species}$ is combined with enzyme $E$ from $S_{enzymes}$ to form a complex $A|E$ and a new reactant $AP$. The choice of kinetic laws used to describe biochemical models depends on the pathway that we will study. In practice, two kinetic laws are often used to describe a biological pathway: the law of mass-action [24] and Michaelis–Menten kinetics [41]. Mass-action kinetics indicate that the rate of a biochemical interaction is proportional to the concentrations of biochemical reactants. A system of nonlinear ODEs defines the rate of change of reactants within experimental time. Thus, an enzyme acts as a catalyst facilitating the reaction in an enzymatic reaction, which is possible to be investigated by biological experiments. Based on literature reviews [64], it is reasonable to use the law of mass-action to model the target biochemical system, which is the RKIP pathway (introduced later in Sect. 5.1). While the law of Michaelis–Menten assumes that the substrate is in instantaneous chemical equilibrium with the complex, and this assumption is not suitable for describing the RKIP pathway. Moreover, the RKIP pathway has been well studied in [13, 21] by using the mass-action law to describe the quantitative relationships between substrates and complex. In order to follow previous research results and develop new modelling methods based on the same biochemical pathway, in this research we also use the mass-action law to describe a pathway and do not consider Michaelis–Menten kinetics at this stage. Although the Michaelis–Menten kinetics are not used in this research, it is possible to use a hybrid method of employing both Michaelis–Menten and mass-action laws to study different parts/scales of biochemical systems as in our previous work [49, 52]. However, this is beyond the scope of this research.

A synthetic enzymatic reaction is shown in Eq. (3). In this equation, the symbol ‘$\rightleftharpoons$’ indicates that the reaction is reversible; the symbol ‘$\rightarrow$’ represents a non-reversible reaction; the symbol ‘$|$’ indicates that a complex reactant is generated from the two reactants (as described before); and the letter $P$ after the species label $A$ means a new product generated from $A$.

3 $$\begin{aligned} A + E \rightleftharpoons A|E \rightarrow AP + E \end{aligned}$$

Therefore, three atomic components can be obtained from the enzymatic reaction shown in Eq. (3): ‘$A + E \rightarrow A|E$’, ‘$A|E \rightarrow A + E$’ and ‘$A|E \rightarrow AP + E$’.

**Fig. 1:**
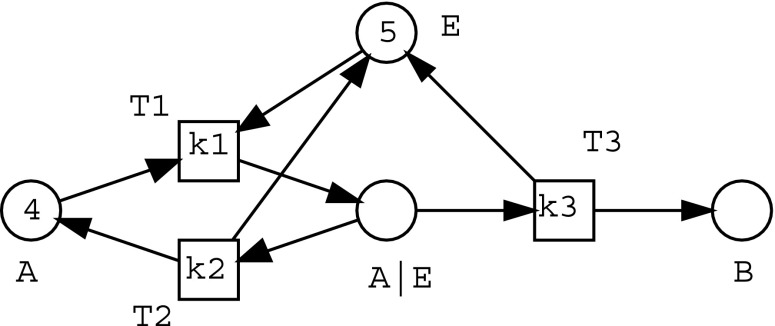
A graphical representation of a Petri net model for an enzymatic reaction consisting of three components

Given a set of reactants, components can be generated from these reactants and used as modules to construct a biochemical model. Components of a model represented by Petri nets are connected by merging the same ‘nodes’ (Places) among these components [66]. Figure 1 shows a Petri net model for an enzymatic reaction consisting of three components connected with each other by merging the same reactants. ‘T1, T2, and T3’ stand for ‘reactions’ (Transitions), whose kinetic rate constants are marked as ‘k1, k2, and k3’ on the places, respectively. The numbers (for example, 4 and 5) associated with the places represent the initial concentrations of reactants [68].

## Qualitative and Quantitative Model Learning Approaches

### Qualitative Model Learning

#### Qualitative States

A dynamic system can be described at a qualitative level, and its important behavioural properties are captured by a set of qualitative states and possible transitions between these states [19, 35]. A qualitative state is a complete assignment of qualitative values to all variables in the system and considered as a ‘snapshot’ of the system. The dynamic system under investigation could potentially demonstrate such possible qualitative states under specific conditions and a correct model built for the system should produce these qualitative states (and only these states if all variables are known).

Table 1 shows a set of qualitative states derived from a qualitative model [71]. Each row in this table represents an individual qualitative state. For each variable, its magnitude and rate of change of the current state are illustrated by the qualitative signs: *pos* (positive), *zer* (zero), and *neg* (negative). For example, if the qualitative value of a variable *A* is $<$*zer*, *pos*$>$, this means the magnitude of *A* is zero and the rate of change is positive, which indicates that the value of *A* is increasing.

**Table 1:** A set of qualitative states

State ID	A	AP	B	BP
1	$\langle$zer , pos$\rangle$	$\langle$pos , neg$\rangle$	$\langle$pos , neg$\rangle$	$\langle$pos , neg$\rangle$
2	$\langle$pos , pos$\rangle$	$\langle$pos , pos$\rangle$	$\langle$zer , pos$\rangle$	$\langle$pos , neg$\rangle$
3	$\langle$pos , zer$\rangle$	$\langle$pos , zer$\rangle$	$\langle$pos , neg$\rangle$	$\langle$pos , neg$\rangle$
4	$\langle$pos , pos$\rangle$	$\langle$pos , pos$\rangle$	$\langle$pos , neg$\rangle$	$\langle$pos , neg$\rangle$
5	$\langle$pos , neg$\rangle$	$\langle$zer , pos$\rangle$	$\langle$pos , neg$\rangle$	$\langle$pos , neg$\rangle$
6	$\langle$zer , pos$\rangle$	$\langle$pos , neg$\rangle$	$\langle$pos , zer$\rangle$	$\langle$pos , neg$\rangle$
7	$\langle$pos , zer$\rangle$	$\langle$pos , neg$\rangle$	$\langle$pos , zer$\rangle$	$\langle$pos , neg$\rangle$
8	$\langle$pos , neg$\rangle$	$\langle$pos , zer$\rangle$	$\langle$pos , zer$\rangle$	$\langle$pos , neg$\rangle$
9	$\langle$pos , zer$\rangle$	$\langle$pos , zer$\rangle$	$\langle$pos , zer$\rangle$	$\langle$pos , neg$\rangle$
10	$\langle$pos , neg$\rangle$	$\langle$pos , pos$\rangle$	$\langle$pos , zer$\rangle$	$\langle$pos , neg$\rangle$
11	$\langle$zer , zer$\rangle$	$\langle$zer , zer$\rangle$	$\langle$zer , zer$\rangle$	$\langle$zer , zer$\rangle$
12	$\langle$pos , pos$\rangle$	$\langle$pos , zer$\rangle$	$\langle$zer , zer$\rangle$	$\langle$zer , zer$\rangle$
13	$\langle$pos , pos$\rangle$	$\langle$pos , zer$\rangle$	$\langle$pos , zer$\rangle$	$\langle$pos , zer$\rangle$
14	$\langle$pos , pos$\rangle$	$\langle$pos , neg$\rangle$	$\langle$pos , pos$\rangle$	$\langle$pos , zer$\rangle$

For the legal transitions between qualitative states, transition rules (e.g., rules presented in QSIM [35]) are employed to calculate them. A sequence of qualitative states forms a *qualitative behaviour*, and the terminal states of the qualitative behaviour are often equilibrium ones, in which all variables remain constant [49].

#### Qualitative Differential Equations

One of the well-studied formalisms of the qualitative model used by QR is qualitative differential equations (QDEs), which have been used by QSIM [33, 35] and Morven [14, 16].

**Fig. 2:**
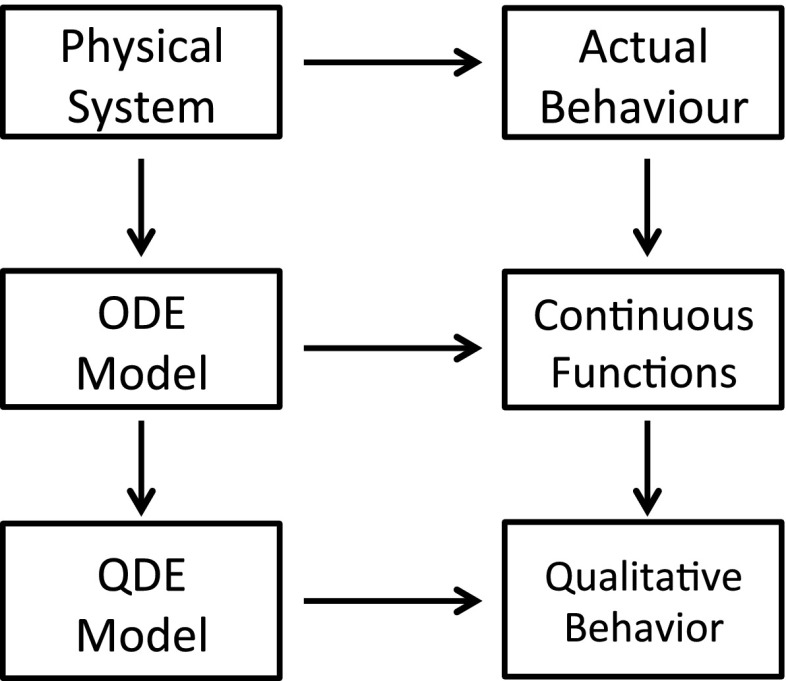
This diagram is a slightly modified version of the one presented in [34]. The diagram shows that all models are abstractions of the real-world systems. Qualitative models are related to ordinary differential equations, but are more expressive of incomplete knowledge

Ordinary differential equations (ODEs) quantitatively describe the behaviour of a dynamic system. A QDE model is the abstraction of a set of ODE models sharing the same model structure but with varying parameter values. Figure 2 shows the relationships between the real-world dynamic systems, ODE models, and QDE models in terms of quantitative and qualitative behaviour.

Formally, a qualitative differential equation (QDE) is a tuple, $<V, Q, C, T>$, each of which is defined in [35] and briefly described as follows: $V$ is a set of qualitative variables, each of which is a ‘reasonable’ function of time; $Q$ is a set of quantity spaces, one for each variable in $V$; $C$ is a set of qualitative constraints applying to the variables in $V$, and each variable in $V$ must appear in some constraints; $T$ is a set of transitions between qualitative states. In summary, a QDE is the conjunction of all its qualitative constraints, and each constraint links some qualitative variables.

According to above definitions, qualitative constraints constitute a QDE, which restricts the generation of possible qualitative states. In addition, a quantity space is composed of several qualitative values that could be taken by a variable. In this research, for the application of ES to evolve the topologies of biochemical models, we use QDEs to represent qualitative models in terms of structure, and for all variables we used the signs quantity space [30], which is composed of three qualitative values: *positive*, *zero* and *negative*.

### Quantitative Model Learning

Quantitative modelling of biochemical processes has been intensively used in biochemical research [1, 7, 12, 55, 61]. Preliminary quantitative analysis of biochemical systems has been very difficult to perform, due to the inherent complexity of biochemical processes [25, 57]. Biochemical systems have been modelled by employing Petri nets theory [44], including enzymatic cascades and synergistic binding of ligands to enzymes [3, 38, 45]. In primary research, the issues of quantitative analysis of metabolic pathways have been studied by Reddy et al. [58, 59]. Research efforts on applying Petri nets to model biochemical processes and the description of current challenges of constructing biochemical pathways by Petri nets are detailed in [4, 11, 39, 56].

In biochemistry, a chemical reaction is a process of converting molecules of reactants known as substrates into products within a specific time period. Biochemical systems are composed of interacting molecular species, whose dynamics are governed by the corresponding chemical reactions. A biochemical model is fully characterised by the initial concentration of each molecular species and the specifications of the reactions with their kinetic rate laws. The dynamics of the molecular species can be described by an ordinary differential equation (ODE) as shown below [67]:

4 $$\begin{aligned} \frac{dX_{i}}{dt} = \sum _{j}\left( \mu _{ij}\cdot \gamma _{j}\prod ^{m}_{k=1}X_{k}^{f_{jk}}\right) , \end{aligned}$$

where $X_{i}$ represents one entity of the model, for instance, the metabolite concentration, protein concentrations, or the level of gene expression; $j$ represents the biochemical reaction that affects the dynamics of the species; $\mu _{ij}$ is the stoichiometric coefficient; $\gamma _{j}$ is the rate constant; $f_{jk}$ stands for kinetic order; and $m$ denotes the number of reactants involved in the reactions.

The use of Petri nets in biochemical systems comes as a natural and intuitive solution, as biochemical reactions are inherently bipartite, and Petri nets can describe relations between biochemical entities [44]: the biochemical reactions and entities can be mapped onto transitions and places, respectively. A continuous Petri net can be represented by a system of ODEs which describes biochemical reactions in models. In this research, we map ODEs from a set of biochemical reactions and optimise the associated kinetic rate constants quantitatively.

## An Integrated and Complementary Biochemical Model Learning Framework

### The Modelling Framework

In this section, we propose an integrated and complementary qualitative and quantitative biochemical modelling framework (2QBMF) for modelling biochemical systems, and this framework can identify the structure and kinetic rate constants simultaneously.

**Fig. 3:**
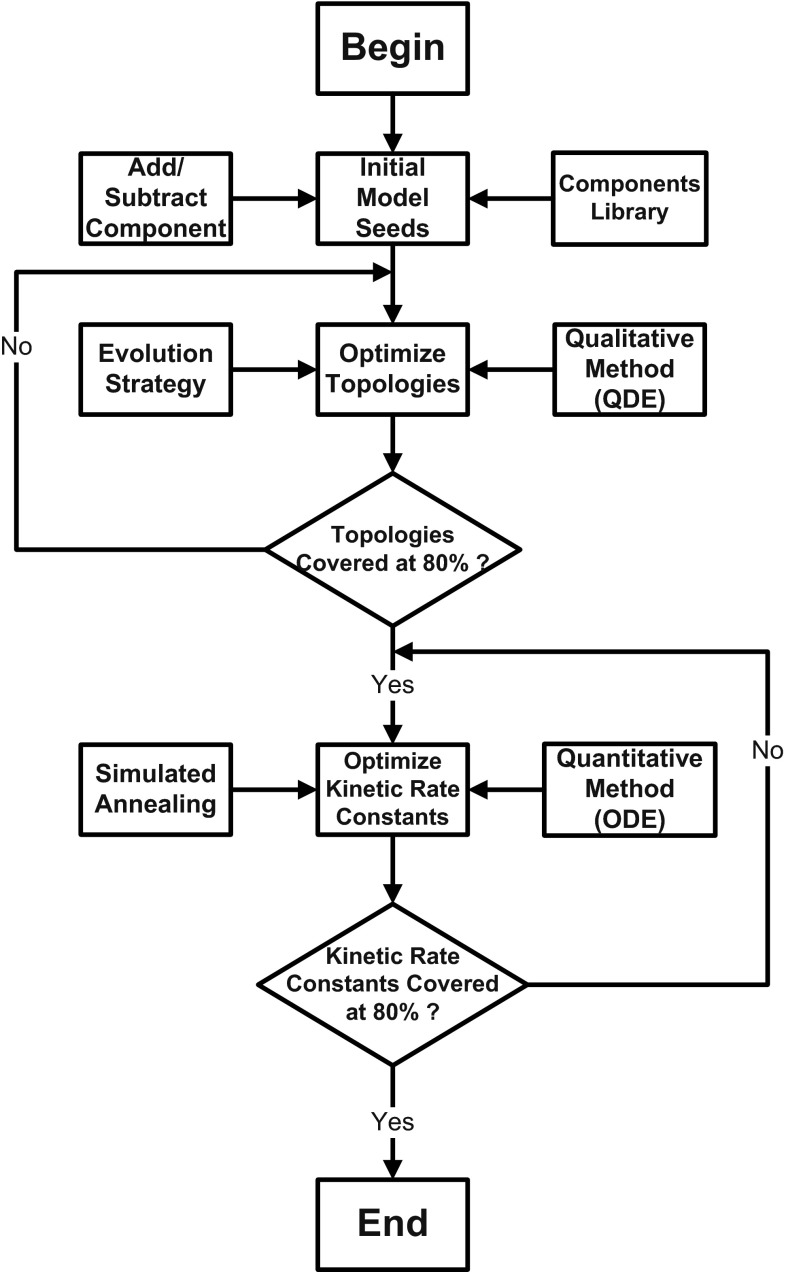
A complementary qualitative and quantitative-based biochemical modelling framework (2QBMF)

Figure 3 illustrates the details of the modelling framework. First, initial biochemical model seeds are synthesised by employing a pair of operators ‘Addition and Subtraction’ to compose biochemical components from a pre-specified component library, before we perform model topological exploration and rate constants optimisation. Qualitative model learning (QML) presented in Sect. 3.1 is then applied to explore the qualitative model space, that is, to explore all possible model structures. As the implicit search space for model structure may be huge, to effectively perform such exploration we use evolution strategy (ES); once we determine the model structure, quantitative model learning (QuatML) described in Sect. 3.2 is employed to optimise the kinetic rate constants, and we use simulated annealing (SA) to perform such optimisation.

While the model structure is finely tuned by the component addition and subtraction operations of ES in an iterative manner, qualitative differential equations (QDEs) converted from the Petri net model are used to describe and analyse the qualitative states generated by simulating each modified model. Modification of model structure is accepted, if the interactions between biochemical reactants in the model can achieve a high rate of coverage (e.g. 80 %), which is defined as the percentage of the observed qualitative states covered by the QDE model.

After the model structure has been explored, kinetic rate constants associated with the biochemical reactions are globally optimised by employing ordinary differential equations (ODEs) in the SA process. The ODEs are performed to mathematically analyse influence of kinetic rate constants on the reactant concentration. If the target rate constants are achieved at a high rate coverage (e.g. 80 %), the optimisation of the reaction rates is accepted.

In this way, the metaheuristic algorithms ES and SA are used to carry out qualitative analysis and quantitative estimation in a complementary manner. The heuristic evolutionary searching mechanism of ES supports the model structure exploration, and the global search strategy of SA helps the optimisation of kinetic rate constants to drive the model behaviours approaching the target ones.

### Qualitative Model Structure Evolution

In the qualitative modelling approach, each individual evolved by ES is a Petri net model. At each generation of the evolutionary process, the topology of the model is evolved through the application of genetic addition and subtraction operators. An evolutionary algorithm ($\mu$+$\lambda$)-ES [5] is employed to iteratively evolve model structure at a qualitative level. In order to test the evolutionary qualitative modelling process in a simplest scenario, we choose a simple (1 + 1)-ES to generate offspring models. Further, advanced ($\mu$+$\lambda$)-ES will be performed and investigated more thoroughly in the future.

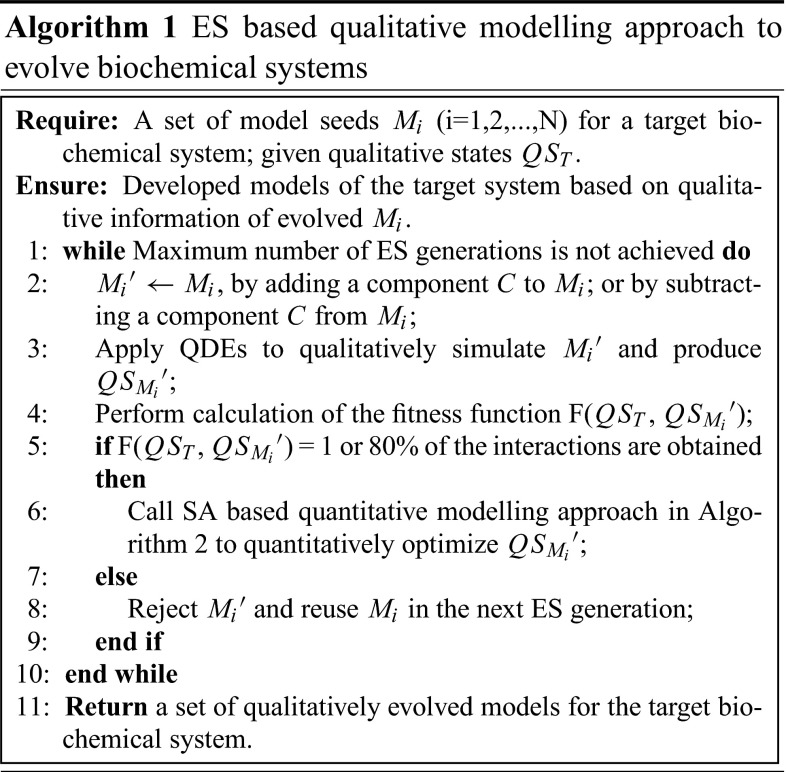



The pseudo-code of employing ES to perform qualitative model learning of biochemical systems is shown in Algorithm 1. A set of model seeds $M_i$ is prepared for structurally approaching the target biochemical system. These model seeds can be obtained from literature or knowledge of wet-laboratory experiments. In general, a composed model ${M_i}'$ can be evolved by applying addition or subtraction operators [66] to $M_i$. In this research, the addition and subtraction operators are applied by a fixed number of generations, which is adopted based on our previous experience in quantitative simulations [67]. Model ${M_i}'$ is simulated with JMorven [8], a qualitative simulation engine. JMorven will generate a set of qualitative states QS for comparison with $QS_T$ in a fitness function F. If the fitness value is equal to 1 (the range of fitness value is from 0 to 1, and the bigger the better) or 80 % of target interactions are obtained (the interactions between reactants in a model indicate the structure of the model), an SA-based quantitative modelling approach in Algorithm 2 will be used to optimise the kinetic rate constants. Otherwise, the mutated ${M_i}'$ is rejected and the mutation is performed in the next generation. The qualitative model learning process is terminated when the ES stopping conditions are satisfied. At the end of the modelling process, a set of final best qualitative models will be obtained with explored topologies by the qualitative analysis.

Note that, one of the predefined conditions, obtaining 80 % of target interactions, is not triggered during the qualitative optimisation process in all simulations performed in this research. The number 80 % was inspired by the Pareto Principle [54] (or called ‘80–20 rule’) which states that roughly 80 % of the effects come from 20 % of the causes for many events. Thus, in our research, it is possible to allow potential good qualitative models to be preserved during the evolutionary process by considering a small group of mutated models with 80 % interactions generated. But after checking our simulation results, we found that the condition of generating 80 % interactions was not hold and switching from Algorithm 1 to Algorithm 2 was always not triggered by this condition. The reason was that fitness values of models under constructions always first reach the value ’1’ before 80 % interactions are generated. However, with an aim to obtain good qualitative models from simulations in further research, we still keep the condition of 80 % interactions as one of criteria for switching from qualitative modelling in Algorithm 1 to quantitative modelling in Algorithm 2.

### Quantitative Model Parameter Optimisation

In the quantitative modelling approach, ODEs are used to mathematically describe biochemical models and parameters of the models are investigated. In this research, kinetic rate values are parameters to be optimised by SA. SA is a heuristic algorithm suitable for globally searching optimal solutions in a very large solution space, and it can avoid getting trapped into local optima. As mentioned in Sect. 3.1.2, a set of ODEs sharing the same structure can be represented by a QDE model; thus, a QDE model obtained from qualitative model learning can be quantitatively optimised to obtain corresponding ODEs. This is achieved by using the QDE model as the skeleton of an ODE model and estimating the parameter values of this ODE model.

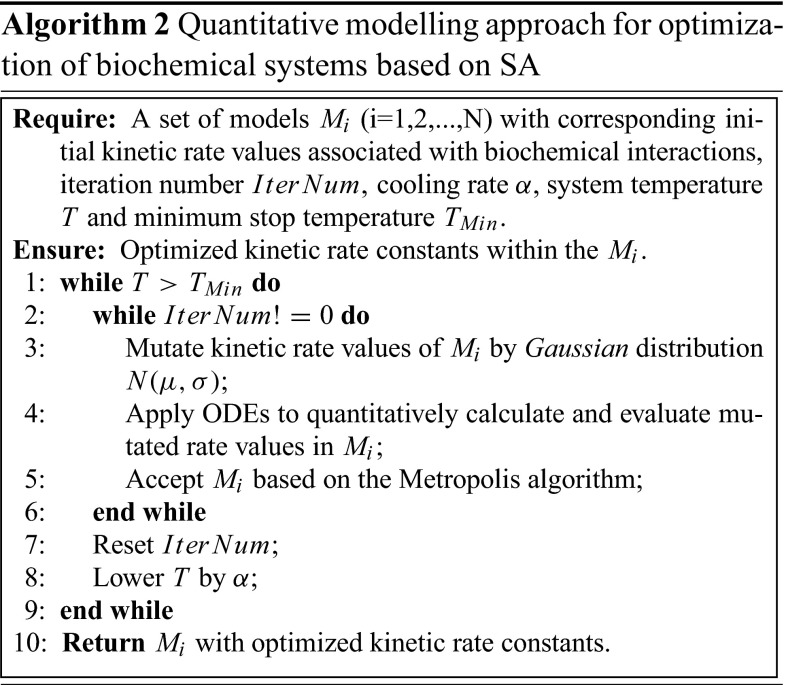



Algorithm 2 shows the pseudo-code of quantitative optimisation of kinetic rates in models $M_i$. Models $M_i$ constructed from previous qualitative model learning stage have been evolved qualitatively to obtain their proper model structures, and these models are to be quantitatively investigated, because the values of the associated rates are essential to obtain expected quantitative behaviours. The parameter $t$ is the current SA system temperature $(t=T)$, and $IterNum$ is the number of iterations at each system temperature.

Models $M_i$ with optimised rate values are accepted or rejected according to a classical Metropolis mechanism [40]. Accepted $M_i$ are preserved as new start points for the next run of model optimisation. Models $M_i$ with different sets of rate values and structures are optimised heuristically at different SA system temperatures by a cooling rate $\alpha$, and the whole process will stop when system temperature reaches the minimum temperature $T_{Min}$.

Note that, due to the probabilistic and random nature of SA [2], a model with a poor estimated fitness value could be generated and accepted. Thus, corresponding biochemical reaction rates associated with different topologies of the models could result in the acceptance of non-optimum models during the model learning process.

### Qualitative and Quantitative Models Evaluation

Models are simulated and evaluated after qualitative structural mutation and quantitative optimisation of rate values. There are two different evaluation methods, one for qualitative and another for quantitative model learning.

#### Qualitative Fitness Function

As mentioned before, qualitative models explored by ES are simulated with JMorven [8]. JMorven can produce a set of qualitative states given a qualitative model. The given target qualitative states of a biochemical system are compared with the states generated from the qualitative model. The number of matched qualitative states between the target system and a synthetic model is recorded and considered as part of the fitness evaluation.

A qualitative state for the model evaluation purpose is an assignment of $N$ variables which appear in both the target biochemical system and explored model. There could be $M$ qualitative states, and a vector is used to record each of these $M$ qualitative states. In this vector, each element is the assignment of one variable. To evaluate a composed model, the following two sets will be compared element by element: one is the set of qualitative states generated by the composed model, and another is the given set of states demonstrated by the target system. In this way, a fitness value of a qualitative model is calculated by considering the overlapping part of the above two sets.

**Fig. 4:**
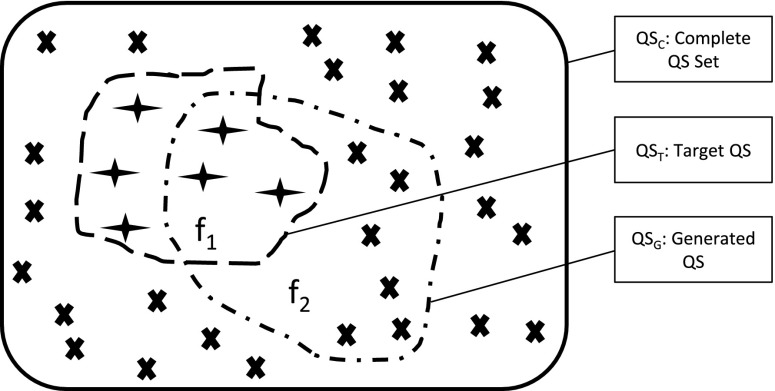
Comparison between generated qualitative states from a synthetic model and target states in a given biochemical system

Figure 4 shows the comparison between a synthetic model and a target biochemical system in terms of qualitative states. A synthetic model produces a set of qualitative states $QS_G$, and a set of given target qualitative states $QS_T$ is compared with $QS_G$ to calculate the coverage of qualitative states in $f_1$. Another comparison is performed by comparing $QS_G$ with the set of all possible states $QS_C$, in which $f_2$ is the rate of matched qualitative states produced by the model under estimation. Therefore, qualitative evaluation of a composed model can be described by jointly considering $f_1$ and $f_2$ in a fitness function $F$. Details of the calculation of $f_1$, $f_2$ and $F$ are shown in Eqs. (5)–(7) [71].

5 $$\begin{aligned} f_1= & {} \frac{\mid QS_G \cap QS_T \mid }{\mid QS_T \mid } \end{aligned}$$

6 $$\begin{aligned} f_2= & {} \frac{\mid QS_G \cap QS_C \mid }{\mid QS_C \mid } \end{aligned}$$

7 $$\begin{aligned} F= & {} 1-\frac{1}{1+f_1+\frac{1}{1+f_2}} \end{aligned}$$

In the above Eqs. (5)–(7), ‘$|\centerdot |$’ denotes the number of states in the set, $\mid QS_G \cap QS_C \mid$ indicates the set of overlapping states in both $QS_G$ and $QS_C$. Two qualitative states are the same if their assignments of all variables are the same.

The value of $f_1$ ranges from 0 (worst) to 1 (best), because the more matched qualitative states between $QS_G$ and $QS_T$, the bigger the value of $f_1$. The value of $f_2$ ranges from 0 (best) to 1 (worse), as the less matched spurious qualitative states between $QS_G$ and $QS_C$, the better the quality of the generated model. A fitness function $F$ is summarised by standardising $f_1$ and $f_2$, and the value of $F$ ranges from 0 (worst) to 1 (best).

Note that there could be different synthetic reactants in a composed model during the evolutionary model learning process, because of the use of genetic addition and subtraction operators. In this research, we specify the reactants to be compared during the model evaluation process. Thus, we discard a composed model of which all reactants are not in the vector of variables for comparison with the target system.

#### Quantitative Fitness Function

Quantitative behaviours of a model are compared with the target ones, which are represented in the form of time series data for the concentrations of species, e.g., enzymes, proteins, and complexes. The behaviours of the species in the target system can be obtained from observations of a biochemical system from the wet laboratory and corresponding computational model in dry laboratory. As mentioned before, the optimisation of kinetic rates will result in the model behaviours approaching the target ones.

A set of reference data $M_T$ is used for the target system, and there are *N* generated time series $X_T=(X_1,X_2,\ldots ,X_N)$ which represent the behaviours of *N* species, and $N \ge 1$. There are *P* data points in each time series $X_i=(x^1_i,x^2_i,\ldots ,x^P_i)^T$, $i=1,\ldots ,N$. There are *M* time series $X_G=(\hat{X}_1,\hat{X}_2,\ldots ,\hat{X}_M)$ which describes the behaviours of *M* species in a constructed model $M_G$, with *P* data points in each time series $\hat{X}_j=(\hat{x}^1_j,\hat{x}^2_j,\ldots ,\hat{x}^P_j)^T$, $j=1,...,M$. Intersection between $M_T$ and $M_G$ is defined by $X_C=X_T \cap X_G=(X_1,X_2,\ldots ,X_n)$, $1 \le n \le N$. The difference between the behaviours of $M_T$ and $M_G$ is calculated by averaging the difference of behaviours of each species in $X_C$ by a paired comparison of the *P* data points.

As shown in Eq. (8), the difference of behaviours for one species $X_k$ in $X_C$ is measured by the *Euclidean* distance, where $\eta$ is the total number of compared substrates in $X_C$ [66].

8 $$\begin{aligned} d_{M_T, M_G}(X_k)=\frac{1}{\eta }\displaystyle \sum _{k=1}^{\eta } \sqrt{\sum _{t=1}^{P}(x_k^t-\hat{x}_k^t)^2} \end{aligned}$$

Note that, $\eta =n$ if the compared substrates are from the intersection $X_C$, ($X_C=X_T \cap X_G$, $|X_C|=n$, $1 \le n \le N$), and $\eta =n'$ if the compared substrates are from a specific $X_C'$, ($X_C' \subseteq X_C$, $|X_C'|=n'$,$1 \le n' \le n$). In this research, quantitative modelling is a minimisation problem; therefore, the smaller the fitness value, the better the generated model.

## Simulation and Analysis

In order to test the effectiveness of our proposed integrative modelling framework, we use a real-world biochemical system, the ‘Ras/Raf-1/MEK/ERK’ signalling pathway [72], which is called ‘The RKIP pathway’ in this research for ease of description. In further research, there will be more key biochemical systems to be used as proof-of-concept examples. Details of the RKIP pathway structure are retrieved from literature as well as experiments, and corresponding qualitative states are abstracted from quantitative values, which are obtained from a simulator Snoopy [27].

We firstly test the feasibility of our learning framework on a small scale: we specify a small number of generations and populations and run experiments with a computer equipped with Intel Core 2 Duo CPU (2.4 GHz) and 4 GB memory; the total number of generations in ES is 100; the number of individual models as model seeds is 20; and in current research, each individual will be mutated definitely without any application of a probability. Thus, individual model is under construction by involving two operators ‘addition’ and ‘subtraction’: addition of one component is performed at each generation, and subtraction of one component is carried out at every two generations which allows model structures to be mutated quickly, according to our previous experience in quantitative simulation [67]; each model seed is an atomic component randomly selected from the component library [66] and applied to the initial population. Various parameter settings of ES and SA (for instance, numbers of generations applying subtraction, addition and crossover in ES; initial temperatures, cooling rates and iteration numbers in SA) have been tested individually throughout the experiments. It appeared that some modelling issues arose as follows on a small scale: if applying the subtraction operator to mutate models more than every five generations, the sizes of model structures would be out of control. If the number of evolutionary generations is more than 100, the simulation process would take a longer time to complete due to the high cost of qualitative and quantitative simulations. If the initial temperature is set too high (e.g. $>100$) and the iteration number is $>50$ in SA, the convergence of model fitness function would be very slow. Therefore, parameter settings of ES and SA in this research are designed according to performance considerations and empirical selection. A comprehensive study of ES and SA parameter settings will be investigated for their effects on a large scale by employing the high-performance computing (HPC) environment in further research. In this way, simulation and analysis on a large scale would present an overall influence of different algorithm parameter settings on the model learning process.

### The RKIP Pathway

Signalling pathways play a pivotal role in many key cellular processes [18]. The abnormality of cell signalling can cause the uncontrollable division of cells, which may lead to cancer. The RKIP pathway is one of the most important and intensively studied signalling pathways, transfers the mitogenic signals from the cell membrane to the nucleus [72]. It is de-regulated in various diseases, ranging from cancer to immunological, inflammatory and degenerative syndromes, and thus represents an important drug target. Ras is activated by an external stimulus, via one of many growth factor receptors; it then binds to and activates Raf-1 to become Raf-1*, or activated Raf, which in turn activates MAPK/ERK Kinase (MEK) which in turn activates Extracellular signal Regulated Kinase (ERK). This cascade (Raf-1 $\rightarrow$ Raf-1* $\rightarrow$ MEK $\rightarrow$ ERK) of protein interaction controls cell differentiation with the effect being dependent upon the activity of ERK. RKIP inhibits the activation of Raf-1 by binding to it, disrupting the interaction between Raf-1 and MEK, thus playing a part in regulating the activity of the ERK pathway.

**Fig. 5:**
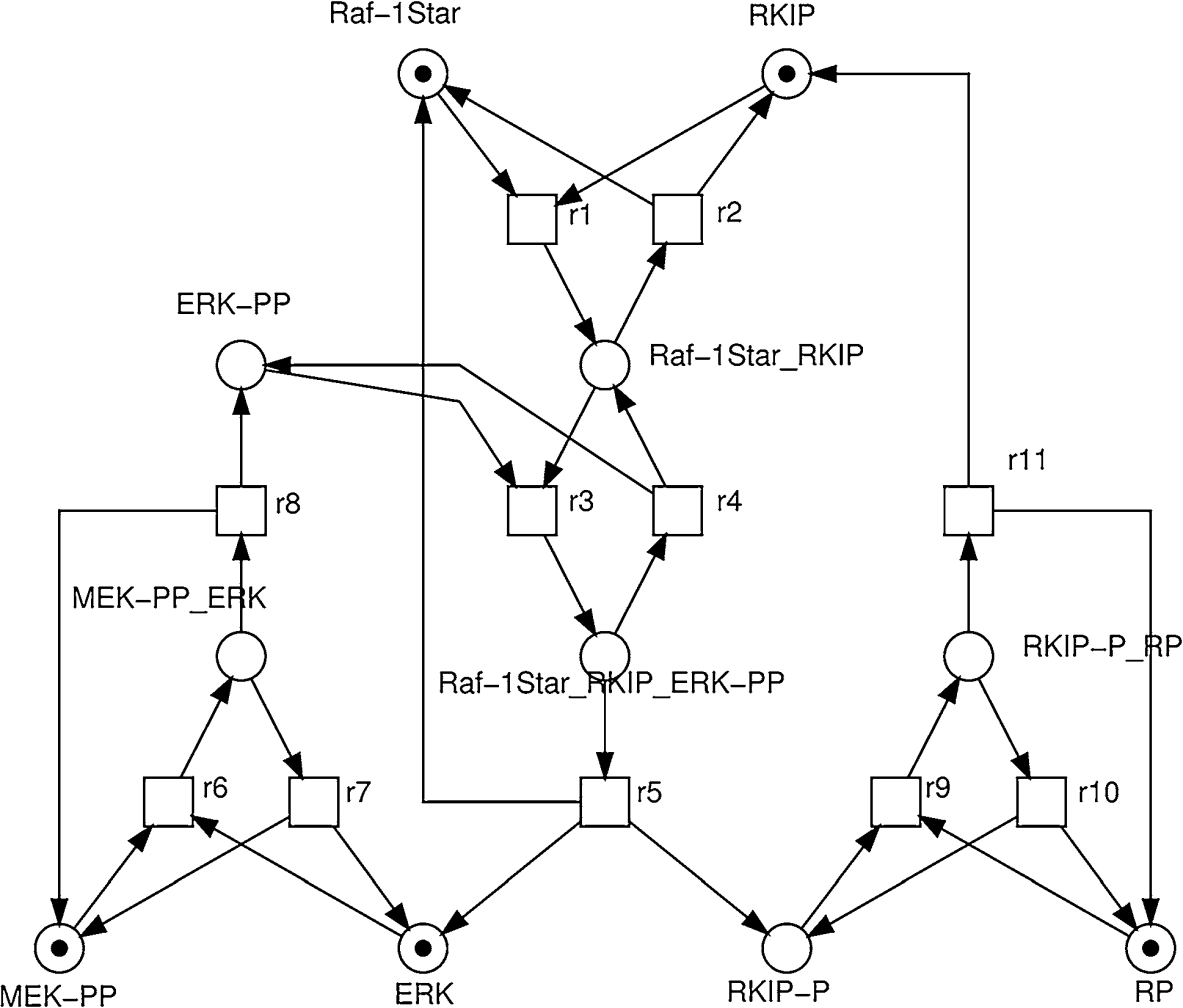
A graphical representation of the ERK signalling pathway regulated by RKIP. Figure is from [21]

With the aim of understanding the role of reactant RKIP in the biochemical pathway, many computational models have been developed and ultimately suggest new therapies [9, 13]. In this research, the RKIP pathway is used to test the proposed modelling framework. Detailed biological description of the RKIP pathway can be found in [13], and a graphical representation of the RKIP pathway in Petri nets from [21] is shown in Fig. 5.

### Analysis of Explored Components

Qualitative model learning focuses on the exploration of components in candidate biochemical models. During the model structure learning process, instantiated components based on given patterns are added into model seeds and components are subtracted from the models. Thus, statistical analysis of the frequency of explored components is important for obtaining information about essential biochemical interactions, the problem of which is very difficult to be addressed in wet laboratory. Moreover, structures of generated models may vary from the target biochemical systems because of the component composition. Regarding the aims of generating interest reactants and hidden complexes associated with the biochemical reactions, it is also helpful to generate synthetic reactants involved in the biochemical models, which can be presented to biologists for further experimental examination.

**Table 2:** Frequency of generated components in models

NO.	Reactants and reactions	Frequency	Target reactions
1	RKIP+Raf1 $\rightarrow$ RKIP$|$Raf1	405	Yes
2	MEKPP+RKIP $\rightarrow$ MEKPP$|$RKIP	407	No
3	RKIPP$|$Raf1 $\rightarrow$ RKIPPP+Raf1	415	No
4	ERK+MEKPP $\rightarrow$ ERK$|$MEKPP	429	Yes
5	ERK$|$MEKPP $\rightarrow$ ERKP+MEKPP	429	No
6	MEKPP$|$RKIPP $\rightarrow$ RKIPPP+MEKPP	433	No
7	RKIP+RP $\rightarrow$ RKIP$|$RP	469	No
8	MEKPP$|$RKIP $\rightarrow$ RKIPP+MEKPP	471	No
9	ERKPP$|$MEKPP $\rightarrow$ ERKPP+MEKPP	483	No
10	MEKPP+RKIPP $\rightarrow$ MEKPP$|$RKIPP	639	No
11	ERKPP$|$MEKPP $\rightarrow$ ERKPPP+MEKPP	761	No

Table 2 shows the top 11 frequently explored components in the qualitatively constructed models. Compared to the structure of the target RKIP pathway, most of these explored components do not exist in the target RKIP pathway:

- Two explored components are in the target RKIP pathway. NO. 1 ‘$RKIP+Raf1 \rightarrow RKIP|Raf1$’ and NO. 4 ‘$ERK+MEKPP \rightarrow ERK|MEKPP$’ components exist in the target RKIP pathway, which means our ES-based qualitative model learning can explore biochemical reactions correctly.
- Other explored components are potential/alternative biochemical reactions in the target RKIP pathway. Most of learned components with high frequency (appearance) are obtained from the analysis of simulation results. After comparing synthetic reactants involved in these components with the target pathway, we can find that alternative biochemical interactions may have influence on the consumption and accumulation of the species concentrations. Thus, this may interest biologists, who can further examine these alternative interactions in wet laboratory.

It is pointed out that functions of these synthetic reactants can be analysed by synthetic biology techniques, which an aim to design new biochemical functionalities for specific tasks in real-world applications.

### Approximating Species Behaviours

Biochemical characteristics of constructed model are described by the changes in species concentrations, which are determined by the biochemical reactions and associated kinetic rates. Quantitative behaviours of the model are represented by time series data, which is from the process of kinetic rates optimisation by SA. Thus, results of kinetic rate optimisation can be examined by comparing the species behaviours between the learned and target models quantitatively.

**Fig. 6:**
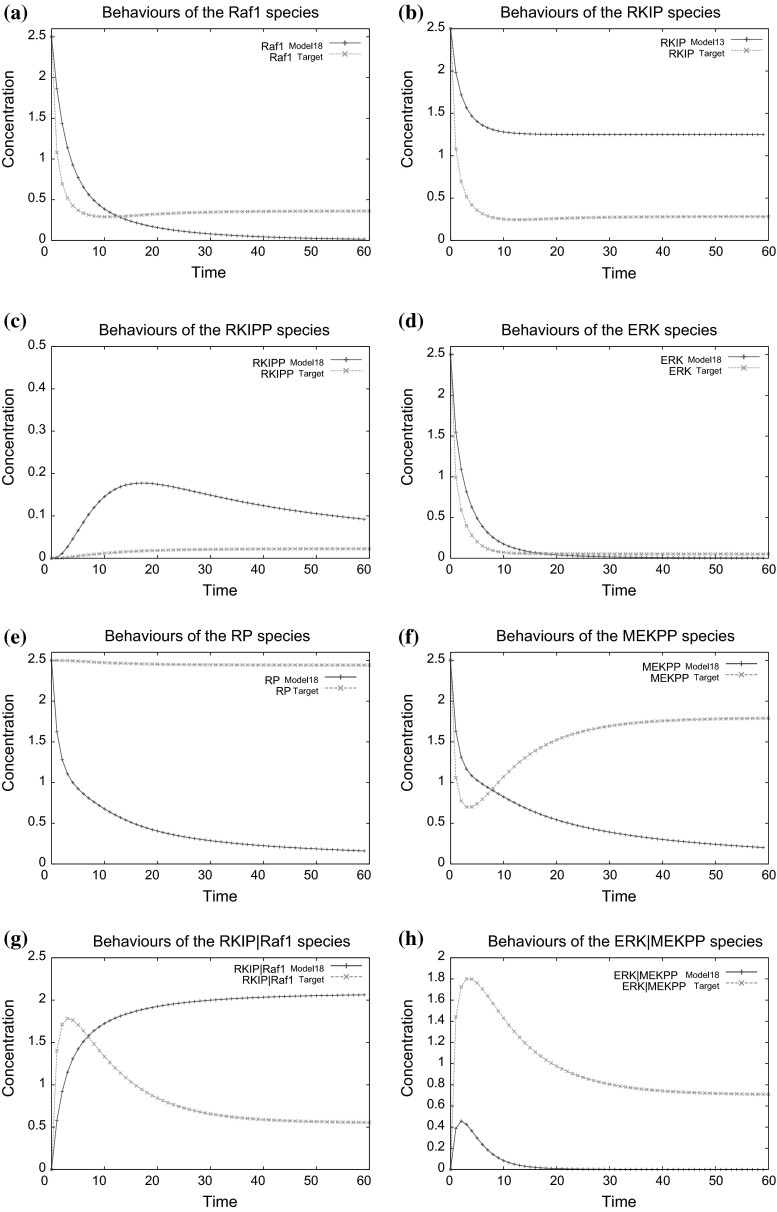
Comparison of species behaviours between one best learned model and the target RKIP pathway **a** Raf1, **b** RKIP, **c** RKIPP, **d** ERK, **e** RP, **f** MEKPP, **g** RKIP|Raf1, **h** ERK|MEKPP

Figure 6 presents the comparison between the behaviours of eight target species in one of the best learned models and those in the target RKIP pathway. From a biologist’s point of view, the ‘shapes’ of species behaviours meaning the qualitative change of species concentrations in experiments are more important than the exact numerical values of species concentrations while performing experiments. Thus, Fig. 6 shows that the behaviours of the learned model qualitatively agrees those demonstrated by the target pathway. This indicates that our proposed model learning framework can effectively optimise the kinetic rates associated with biochemical reactions, which in turn drive the species behaviours of the synthetic models to approach the target species behaviours in an approximate manner.

In future study, when performing quantitative optimisations of associated kinetic rate values on a large scale, dissimilarities of these molecular species behaviours could be further reduced by increasing the number of obtained reactants and interactions between these reactants. Another way of driving the shapes of molecular species behaviours is to generate appropriate interactions between target molecular species and hidden complex in the evolved models at optimised kinetic rate values.

## Comparison with Relevant Systems

There are a number of biochemical modelling frameworks which consider both qualitative and quantitative perspectives.

For instance, Guerriero has used the gp130/JAK/STAT signalling pathway as a case study for modelling and performs analysis using the Bio-PEPA process algebra in [23]. In that work, the PRISM model-checker [28] was used to verify a number of qualitative properties of a model generated by the Bio-PEPA Workbench. The qualitative properties of a PRISM model include deadlock states, species invariants, reachability, reversibility and liveness. These properties are intended to be consistency checks on the model, which allow the presence of possible human errors to be checked in the modelling process and the model can be guaranteed to behave as expected. Then, the PRISM model-checker was used to perform quantitative analysis: state reward-based properties were considered to observe the time series for some of the quantitative species of the system; additional (semi-)quantitative measures were also defined for further quantitative computation.

Milazzo [42] developed a formalism for the description of biological systems, called Calculus of Looping Sequences (CLS), based on term rewriting. Some typical features of process calculi for concurrency were included in CLS. Formalisms were introduced for the description of biological systems and formal tools were provided for the verification of properties of biological systems. Qualitative aspects of biological systems (structure and presence/absence of certain molecules) were also considered, concluding that it is only possible to verify qualitative properties such as the reachability of particular states or causality relationships between biological events. Moreover, a stochastic extension of CLS (Stochastic CLS) was developed, in which quantitative aspects of biological systems such as time and probabilities were taken into account.

However, the above methods did not suggest how to apply both quantitative and qualitative methods and their integration to optimise the candidate models in terms of their structure and kinetic rates. Our learning framework focuses on the optimisation of structure and kinetic rates within the same framework, and we employ Petri nets to represent models and QML to infer and verify qualitative models. These features make our approach distinguish from the above-mentioned approaches.

Finally, it is worthwhile to investigate the application of other evolutionary algorithms and soft computing techniques in our integrative learning framework. Therefore, it will be interesting to compare the performance of ES and SA with that of other evolutionary algorithms and soft computing techniques when they are applied to our learning framework. In future research, we plan to employ classical bio-inspired algorithms, for instance, the genetic algorithm (GA) and ant colony optimisation (ACO) algorithm, in our learning framework to investigate whether these algorithms are suitable for our learning tasks or could improve the overall learning performance.

## Conclusions

The lack of biochemical knowledge and the limitation of experimental techniques are reasons that studying biochemical systems in wet laboratory is a time-consuming and expensive task. Modelling biochemical systems in silico helps the investigation of biochemical systems in nature. Thus, it is important and interesting for life scientists to use alternative routes to study biochemical systems. Existing modelling approaches are either qualitative when there are only sparse, noisy data and incomplete knowledge available, or quantitative when there are sufficient reliable quantitative data. However, in many circumstances, the model structure for the underlying system and associated reactants may not be well identified due to the problem nature, the lack of data and knowledge, and technical limitations. Therefore, there is a need to perform model identification at both qualitative and quantitative levels, that is, qualitatively explore the model structure space (the topology of a model) and quantitatively optimise the parameters of the target biochemical systems in an integrative manner.

In this research, we show how the identification of biochemical systems can be performed and evolved in an integrative manner by reusing, composing, and evolving biochemical modules qualitatively and by mutating kinetic rates quantitatively. The main issues of the integrative qualitative and quantitative model learning are addressed in this research: firstly, interactions between reactants (including potential reactants to be discovered) are learned by the qualitative model learning approach with an evolutionary algorithm; secondly, kinetic rates in a generated biochemical model are quantitatively optimised so that behaviours of the target biochemical systems are reproduced in synthetic models. Experimental results have shown that our proposed integrative qualitative and quantitative model learning framework is feasible and effective in the presence of incomplete knowledge and qualitative data. We point out that our model learning framework can be applied in both the context of computational systems biology for biochemical system identification and synthetic biology for the modular design of desired biochemical systems.

In future research, a high-performance computing environment will be used to improve the learning performance of our framework. By performing parallelised construction of biochemical models, for instance, models for metabolic or signalling pathways participated by some key species, much more biochemical meaningful models can be obtained and further investigated by biologists in wet laboratory. Furthermore, after generating a large number of biochemical models, some biochemical interaction patterns can be statistically analysed and summarised for later reuse as modules for piece-wise model composing [68]. Another interesting research direction is to investigate the effectiveness of more established and state-of-the-art heuristic algorithms, especially evolutionary algorithms, when they are applied to our integrative qualitative and quantitative system identification tasks. Finally, we expect that our learning framework can be implemented as a user interactive program for biologists so that they can make use of the program to facilitate experiments and generate feedbacks.
